# Comparison of the ocular tolerability of a latanoprost cationic emulsion versus conventional formulations of prostaglandins: an in vivo toxicity assay

**Published:** 2009-08-25

**Authors:** Hong Liang, Christophe Baudouin, Marie-Odile Faure, Grégory Lambert, Françoise Brignole-Baudouin

**Affiliations:** 1 Paris Descartes University, Faculty of Biological and Pharmacological Sciences, Department of Toxicology, Paris, France; 2 INSERM, UMR_S968, Institut de la Vision, department of Therapeutics, Paris, France; 3 UPMC University Paris, Institut de la Vision, Paris, France; 4 Quinze-Vingts National Hospital of Ophtalmology - Department of Ophthalmology, Paris, France; 5 Quinze-Vingts National Hospital of Ophtalmology - Department of Biology, Paris, France; 6 Ambroise Paré Hospital, APHP, Versailles Saint-Quentin en Yvelines University, Paris, France; 7 Novagali Pharma SA, Evry, France

## Abstract

**Purpose:**

Using an established rabbit toxicological model, this in vivo study compared the ocular cytotoxicity of four topical intraocular pressure (IOP)-lowering agents: the commercial benzalkonium chloride (BAC)-containing solutions of 0.005% latanoprost, 0.004% travoprost, 0.03% bimatoprost (containing 0.02%, 0.015%, and 0.005% BAC, respectively), and 0.005% latanoprost in a new cationic emulsion (LCEm) formulation.

**Methods:**

Thirty adult male New Zealand albino rabbits were used in this study. They were randomly divided into five groups: 50 µl of sterile phosphate-buffered saline (PBS) along with each formulation was applied onto rabbit eyes 15 times at 5 min intervals. The ocular surface changes were investigated using slit-lamp examination, corneal in vivo confocal microscopy (IVCM) for cornea, limbus, conjunctiva/conjunctiva-associated lymphoid tissue (CALT) investigations, and conjunctival imprints for cytology and flow cytometry (FCM) analyses.

**Results:**

Antiglaucoma eye drops induced an ocular surface cytotoxicity primarily related to the concentration of their common BAC preservative (^0.02%BAC+^latanoprost> ^0.015%BAC+^travoprost> ^0.005%BAC+^bimatoprost). LCEm did not induce any obvious signs of toxicity on the rabbit ocular surface with results similar to those of PBS; moreover, the conjunctiva/CALT and cornea had almost normal aspects.

**Conclusions:**

These in vivo and ex vivo toxicological procedures performed in an acute stress model confirmed the ocular surface cytotoxicity of BAC-containing antiglaucomatous eye drop solutions. The new formulation, LCEm, was well tolerated without inducing ocular surface damage or CALT activation. The cationic emulsion of latanoprost will most likely have fewer long-term adverse effects on the ocular surface than formulations containing toxic preservative BAC and may improve long-term tolerance over BAC-containing antiglaucomatous topical treatments.

## Introduction

Glaucoma remains the second leading cause of blindness in the world [1], and an estimated 60.5 million people worldwide will be affected by open-angle glaucoma and angle-closure glaucoma in 2010, increasing to 79.6 million by 2020 [2]. While the efficacy of a neuroprotective strategy remains unclear, it has been shown that intraocular pressure (IOP)-lowering agents, such as prostaglandin (PG) analogues, play a major role in glaucoma treatment [3].

Today, long-term antiglaucoma eye drop therapy requires not only efficacy and safety, but also good tolerability for improved patient comfort, and hence better compliance. However, the toxic preservative benzalkonium chloride (BAC) is still an important excipient found in the vast majority of PG eye drop formulations. BAC is a mixture of alkylbenzyldimethylammonium chlorides used for the bactericidal and microbicidal activity of its C12 and C14 alkyl derivatives [4]. Numerous in vitro and ex vivo studies have reported cytotoxic effects of the major IOP-lowering agents containing BAC [5-9]. For example, BAC-containing latanoprost (^BAC+^latanoprost) and timolol (^BAC+^timolol) eye drops exhibited higher proinflammatory and proapoptotic effects on conjunctival cells than did BAC-free timolol (^BAC-^timolol) eye drops [9]. In rat corneal and conjunctival epithelial cells, stress-related genes (c-*fos* and c*-jun*) were transcriptionally activated and the cyclooxygenase-2 (*COX-2*) gene was overexpressed after BAC-containing antiglaucoma medications, such as ^BAC+^timolol, ^BAC+^latanoprost, or ^BAC+^isopropyl unoprostone treatment [10].

Nowadays, the ocular cytotoxicity of antiglaucoma treatment can be greatly reduced by the use of preservative-free single-dose units, such as carteolol [11] or tafluprost [12,13], and preserved BAC-free multi-dose systems, such as the ABAK system available for nonpreserved beta-blockers, or self-preserved ionic-buffered system (e.g., sofZia^®^) found in Travatan Z^®^ solution. Compared to BAC-preserved eye drops, preservative-free eye drops consistently induced significantly fewer ocular symptoms and signs of irritation in patients, such as pain or discomfort, foreign body sensation, stinging or burning, and dry eye sensation [14]. Many in vitro or ex vivo studies were conducted to demonstrate their good tolerance [6,9,12]. Cationic micro-emulsions are another interesting approach, offering a new way to deliver lipophilic drugs to the ocular surface [15-17]. These cationic emulsions, through the electrostatic attraction of their positively charged oil droplets with the highly negatively charged ocular surface mucins, have an increased ocular surface residence time, hence improving the absorption of the active ingredient loaded in the emulsion. This new technology has been successfully developed for the treatment of mild dry eye symptoms (Cationorm®) [18,19]. This very similar cationic emulsion used as a carrier for cyclosporine A has entered a phase II clinical trial in the United States and a phase III clinical trial in Europe for the treatment of Sjögren's syndrome and vernal keratoconjunctivitis, respectively [20,21]. In a previous animal study [19], we demonstrated that nonpreserved cationic emulsion vehicles are very well tolerated by the ocular surface and induced no cytotoxicity, even after repeated applications [19]. This interesting new technology can also be used for the efficient delivery of antiglaucoma PG analogues. Moreover, as these cationic emulsions are devoid of any preservative effect, they have a better ocular surface tolerance than standard PG analogue formulations, as a result of BAC removal.

The aim of this in vivo study was to assess the ocular tolerance of the prostaglandin analogue latanoprost, in a newly developed cationic emulsion formulation (LCEm), and to compare it with more classic BAC-containing antiglaucoma eye drops: ^0.02%BAC+^latanoprost, ^0.015%BAC+^travoprost, and ^0.005%BAC+^bimatoprost. In this study, we compared these PGs using clinical observations, analyses of microstructures of the ocular surface by in vivo confocal microscopy (IVCM), and impression cytology (IC). None of these in vivo and ex vivo methods involved animal sacrifice, and they provided standardized cell-level analyses by quantified systems. In addition, we were particularly interested in investigating the conjunctiva-associated lymphoid tissue (CALT), a structure that plays an important role in the innate and acquired ocular surface immunity. Careful analysis of the inflammatory markers within the CALT allowed us to precisely discriminate the different PG-containing antiglaucoma eye drops with regard to their specific ocular toxicity.

## Methods

### Animals

All experiments were conducted in accordance with the ARVO Statement for the Use of Animals in Ophthalmic and Vision Research. Thirty male New Zealand white (NZW) rabbits were randomly divided into five groups: each group consisted of six rabbits for clinical evaluation, IVCM observation, and conjunctival imprint collection at the time points of 4 h (H) and 1 day (D; three rabbits for H4 and three for D1). The time points were chosen according to a previous study [19].

### Antiglaucoma eye drop treatments

Fifty microliters of treatment preparation was instilled according to a previous toxic model [19,22]: sterile phosphate-buffered saline (PBS), three commercial solutions of 0.005% latanoprost containing 0.02% BAC (Xalatan®; Pfizer, New York, NY), 0.004% travoprost containing 0.015% BAC (Travatan®; Alcon, Fort Worth, TX), 0.003% bimatoprost containing 0.005% BAC (Lumigan®; Allergan, Irvine, CA), and a new formulation of 0.005% latanoprost in a cationic emulsion (LCEm; Novagali Pharma, Evry, France). The LCEm was nonpreserved with physiological pH and osmolality, and contained 0.002% cetalkonium chloride (CKC) acting as a cationic agent. In this model of acute toxicological stress, each solution was applied onto the ocular surface 15 times, at 5 min intervals, with a sterile micropipette.

### Clinical findings and Draize test

The first instillation was chosen as time zero (T0). At H4 and D1 the eyes were examined using a slit-lamp. Ocular irritation was scored according to a modified Draize test [19].

### In vivo confocal microscopy observation and new IVCM-CALT scale

A laser-scanning IVCM Heidelberg Retina Tomograph (HRT) II/ Rostock Cornea Module (RCM; Heidelberg Engineering GmbH, Heidelberg, Germany) was used to examine the entire ocular surface (cornea/conjunctiva/limbus) as previously described [19]. An IVCM scale system was used as in a previous study [13].

The CALT structure was examined as previously described by our team [23]. Based on the published IVCM scale [13,24], we developed a new IVCM scale including the CALT structure description in order to quantify the ocular surface reactions more extensively (Table 1). We especially took into account the levels of inflammatory cell infiltration at the periphery (outside) and in the center (inside) of the CALT follicles. We scored up to four levels of inflammatory cell infiltration: (0/1); no or very slight infiltration (0–100 inflammatory cells/mm^2^), (2); moderate (100–500 inflammatory cells/mm^2^), (3); pronounced (500–1000 inflammatory cells/mm^2^), and (4); severe (>1000 inflammatory cells/mm^2^).

**Table 1:** In vivo confocal microscopy scoring for the evaluation of ocular toxicity in CALT analysis.

**CALT reactivity is evaluated in two different areas: inside and outside the follicle**	
Inflammatory cell infiltration score	Inflammatory cells/mm^2^
0/1	0–100
2	100–500
3	500–1000
4	>1000

For IVCM-CALT analysis, we took into account the level of inflammatory cell infiltration at the periphery (outside) and in the center (inside) of the superficial layers in CALT follicles: (0/1): no or very slight infiltration (0–100 inflammatory cells/mm^2^); (2): moderate (100–500 inflammatory cells/mm^2^); (3): pronounced (500–1000 inflammatory cells/mm^2^), (4): severe (>1000 inflammatory cells/mm^2^).

### Conjunctival impression cytology analysis for cytology and flow cytometry analyses

Impression cytology specimens were collected from the superior conjunctiva using techniques previously described [19]. Two types of filter paper were used: two nitrocellulose membranes (Millipore, Bedford, MA) were applied to the superior bulbar conjunctiva for cresyl violet cytology (1%, number 5235; Merck, Fontenay-sous-Bois, France), and two polyether sulfone membranes (Supor®; Gelman Sciences, Ann Arbor, MI) were prepared for flow cytometry (FCM) procedures.

For the cresyl violet staining, cellular damage and inflammatory cell infiltration were quantified according to a published IC score system [25]. Conjunctival cells were also extracted and analyzed using FCM (FC500; Beckman Coulter, Miami, FL). Indirect immunofluorescence procedures were used for CD45-positive hematopoietic cell staining (1:50; MCA808G; Serotec AbD, MorphoSys, UK), and the results were expressed as percentages of positive cells. After the FCM analysis, the cell suspension was stained with propidium iodide (PI 0.5 µg/ml; Sigma Chemical Company, St. Louis, MO), and was spun down on a glass slide using a cytospin centrifuge (Shandon Cytospin 4; Thermo, Electron Corporation, Waltham, MA) for analysis with a confocal microscope (E800; PCM 2000; Nikon, Tokyo, Japan).

### Statistical analysis

All statistical comparisons were performed with two-way analysis of variance (ANOVA), followed by multiple pair-wise comparisons using Fisher’s method adjustment (Statview V for Windows; SAS Institute, Cary, NC).

## Results

### Clinical observation and Draize test

**Figure 1:**
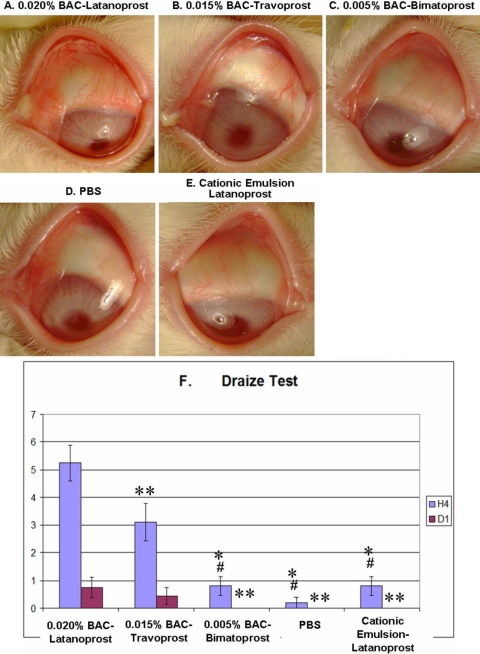
Microphotographs of typical clinical features after 15 instillations of different prostaglandins and Draize test evaluation at 4 h. Microphotographs of typical clinical features of instilled rabbit eyes at H4 for ^BAC+^latanoprost- (**A**), ^BAC+^travoprost- (**B**), ^BAC+^bimatoprost- (**C**), PBS- (**D**), and latanoprost in cationic emulsion (LCEm)-treated groups (**E**). ^BAC+^Latanoprost and ^BAC+^travoprost induced diffuse hyperemia, chemosis, and purulent secretions on the ocular surface. ^BAC+^Bimatoprost also induced mild conjunctiva hyperemia. LCEm-receiving eyes only presented slight hyperemia. Bar chart of ocular irritation evaluations using a modified Draize test scale (**F**) at H4 and D1. The asterisk indicates a p<0.0001 compared with the ^BAC+^latanoprost-instilled group. The double asterisk indicates a p<0.01 compared with the ^BAC+^latanoprost-instilled group, and the sharp (hash mark) indicates a p<0.001 compared with the ^BAC+^travoprost-instilled group.

After instillations, all three BAC-containing PGs induced an obvious ocular surface toxicity in rabbits: ^BAC+^latanoprost (Figure 1A) and ^BAC+^travoprost (Figure 1B) induced obvious hyperemia, chemosis, and purulent secretions; ^BAC+^bimatoprost (Figure 1C) induced mild hyperemia but with no secretions. PBS (Figure 1D) presented a normal ocular surface aspect, while LCEm (Figure 1E) presented only slight hyperemia.

At H4, the ^BAC+^latanoprost-treated animals had the highest Draize score compared to all other groups (p<0.01 versus ^BAC+^travoprost, and p<0.0001 versus the remaining three groups; Figure 1F). ^BAC+^Travoprost-treated rabbits had higher Draize scores than those treated with ^BAC+^bimatoprost, PBS, or LCEm (p<0.001 for three groups), without any differences among the last three groups. At D1, the Draize score for the ^BAC+^latanoprost group remained the highest (p<0.01 when compared with ^BAC+^bimatoprost, PBS and LCEm groups). At D1, in the ^BAC+^travoprost group, the treated eyes had not yet returned to their normal aspect, but there were no statistical differences when compared with the other groups (p>0.05). The ^BAC+^bimatoprost, PBS, and LCEm groups all presented normal aspects at D1.

### IVCM images and two IVCM scale analyses

#### Superficial epithelium

**Figure 2:**
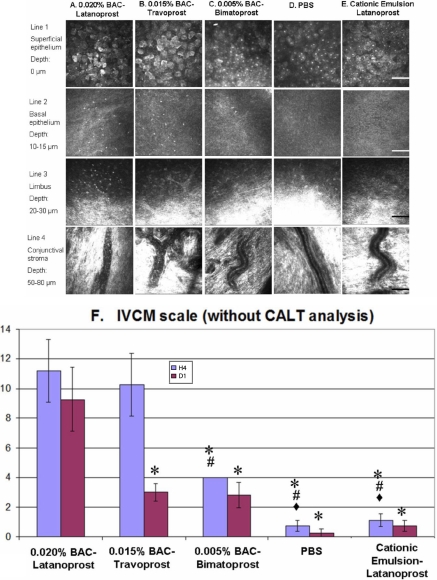
IVCM images of rabbit ocular surface (cornea, limbus, and conjunctiva). IVCM images of rabbit ocular surface at H4 after ^BAC+^latanoprost (**A**), ^BAC+^travoprost (**B**), ^BAC+^bimatoprost (**C**), PBS (**D**), and LCEm (**E**) instillations, of the superficial epithelium (line 1), the basal epithelium (line 2: 10–15 μm from the superficial epithelium layer), the limbus (line 3: about 20–30 μm from the superficial epithelium layer), and the conjunctival substantia propria (line 4: 50–80 μm from the superficial epithelium layer). ^BAC+^Latanoprost and ^BAC+^travoprost-treated eyes showed the greatest damage in the epithelium and the greatest inflammatory cell infiltration in the basal epithelium and limbus. ^BAC+^Bimatoprost induced slight inflammation in the basal epithelium. These three BAC-containing eye drops induced inflammatory cells rolling in conjunctival blood vessels. PBS and LECm did not induce any obvious ocular surface microstructure damage. The scale bar indicates 100 μm. IVCM scores (**F**) for the five tested groups. ^BAC+^Latanoprost and ^BAC+^travoprost presented the highest IVCM toxic score at H4 and D1, with intermediate results for ^BAC+^bimatoprost. The toxicity of LECm was less than that of the three BAC-containing commercial prostaglandins with no significant difference with the PBS group at all time points. The asterisk indicates a p<0.002 compared with ^BAC+^latanoprost. The sharp indicates a p<0.002 compared with ^BAC+^travoprost and the filled diamond indicates a p<0.05 compared with ^BAC+^bimatoprost.

At H4, ^BAC+^latanoprost (Figure 2A; line 1) and ^BAC+^travoprost-treated eyes (Figure 2B; line 1) displayed various abnormalities of the corneal epithelium, including partial desquamation, irregular cell shapes, abnormal reflectivity patterns, swollen cells, and occasional inflammatory cell infiltration. ^BAC+^Bimatoprost (Figure 2C; line 1) did not induce such major changes, with only rare irregular cell shapes observed. PBS- and LCEm-instilled (Figure 2D,E; line 1) rabbits presented eyes with normal aspects.

#### Basal epithelium

^BAC+^latanoprost (Figure 2A; line 2), ^BAC+^travoprost (Figure 2B; line 2), and ^BAC+^bimatoprost (Figure 2C; line 2) all induced inflammatory cell infiltration at levels related to their concentrations of the preservative BAC: 126.50±16.89 cells/mm^2^ for ^BAC+^latanoprost (p<0.01 when compared with all the other groups); 89.70±10.72 cells/mm^2^ for ^BAC+^travoprost (p<0.01 when compared with the other groups); and 21.40±3.11 cells/mm^2^ for ^BAC+^bimatoprost. PBS and LCEm (Figure 2D,E; line 2) did not induce any inflammation (<5 cells/mm^2^) during the entire observation time.

#### Limbus

For the ^BAC+^latanoprost group (Figure 2A; line 3), obvious inflammatory cell infiltration was noted in the peripheral cornea and limbus area. No obvious reactions were seen in the other groups.

#### Conjunctival blood vessels

For all BAC-containing PG-treated groups (Figure 2A,B,C; line 4), a phenomenon of inflammatory cell rolling was noted, as characteristic cells fixed alongside the blood vessel wall were observed. The PBS and LCEm groups (Figure 2D,E; line 4) did not induce such obvious rolling of inflammatory cells.

#### IVCM scale

The whole ocular surface cytotoxicity was scored by using the IVCM scale (Figure 2F). At H4, treatment with ^BAC+^latanoprost and ^BAC+^travoprost eye drops induced the highest IVCM scores when compared with the other groups (p<0.002 for both). ^BAC+^Bimatoprost induced moderate toxicity by IVCM analysis, which was lower than ^BAC+^latanoprost and ^BAC+^travoprost (p<0.002), but still higher than PBS and LCEm (p<0.05). The LCEm-treated group was not statistically different from the PBS-instilled group. At D1, the IVCM score remained very high after ^BAC+^latanoprost application (p<0.002 when compared with the four other groups). In contrast, the IVCM scores of the other four groups decreased, with no statistical difference among them at D1 (p>0.1).

#### Conjunctiva-associated lymphoid tissue

**Figure 3:**
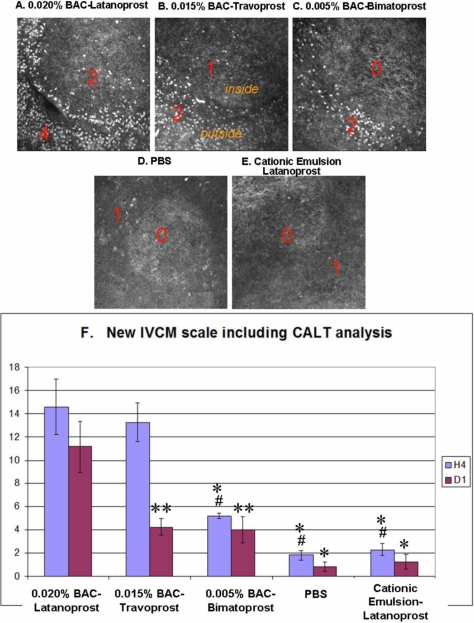
IVCM images of rabbit CALT after instillations with different PG eye drops. IVCM images of rabbit CALT at H4 after ^BAC+^latanoprost (**A**), ^BAC+^travoprost (**B**), ^BAC+^bimatoprost (**C**), PBS (**D**), or LCEm (**E**) instillations. Numerous inflammatory cells were observed infiltrating the periphery and center of the CALT structure, especially after the instillation of ^BAC+^latanoprost. LCEm did not induce any obvious inflammatory cell infiltration, as in the PBS-instilled eyes. The scale bar indicates 100 μm. New IVCM scale including CALT description shows the following toxicity order (**F**): ^BAC+^latanoprost > ^BAC+^travoprost > ^BAC+^bimatoprost at H4. There was no significant difference between the PBS and LCEm groups at all times. The asterisk indicates a p<0.0001 compared with ^BAC+^latanoprost. The double asterisk indicates a p<0.003 compared with ^BAC+^latanoprost and the sharp indicates a p<0.0002 compared with ^BAC+^travoprost.

Figure 3 shows representative IVCM images of the superficial layers of CALT follicles at H4. Following the instillation of ^BAC+^latanoprost and ^BAC+^travoprost eye drops, severe inflammatory cell infiltration was observed, especially in the follicle and parafollicular areas. This infiltration consisted of numerous hyper-reflective patterns in the ^BAC+^latanoprost (Fig. 3A) and ^BAC+^travoprost (Figure 3B) groups corresponding to polymononuclear leukocytes (PMNs), lymphocytes, and dendritiform cells as demonstrated by a previous immunohistology study [23]. Application of bimatoprost eye drops (Figure 3C) containing 0.005% BAC also induced a moderate infiltration of hyper-reflective inflammatory cells around the CALT follicle. In contrast, instillation of PBS (Figure 3D) did not induce active changes in the CALT structure. Similarly, when LCEm was instilled onto the ocular surface, no special activation was found (Figure 3E). The inflammatory cell infiltration decreased dramatically but still persisted at D1 for three BAC-containing groups (data not shown).

#### New IVCM scale including CALT analysis

To increase the resolution power of the IVCM, we decided to include the CALT activation data into the aforementioned IVCM scale, and create a new IVCM scale (IVCM + CALT). This new IVCM scale follows the same trend as our previously published IVCM scale (Figure 3F). At H4, the ^BAC+^latanoprost and ^BAC+^travoprost groups had higher toxicity scores than the other three groups (p<0.0001 for ^BAC+^latanoprost, p<0.0002 for ^BAC+^travoprost). ^BAC+^Bimatoprost also induced a moderate toxic score, which was lower than two other BAC-containing eye drops but still higher than PBS and LCEm groups. The LCEm group scores still showed no significant statistical difference to the scores from the PBS group. At D1, ^BAC+^latanoprost-treated rabbits still had the highest IVCM score (p<0.003 when compared with ^BAC+^travoprost- and ^BAC+^bimatoprost-treated groups; p<0.0001 when compared with the PBS and LCEm groups). The scores decreased in the other four groups between H4 and D1, and presented no statistical differences among them.

### IC staining, IC score and FCM analysis

**Figure 4:**
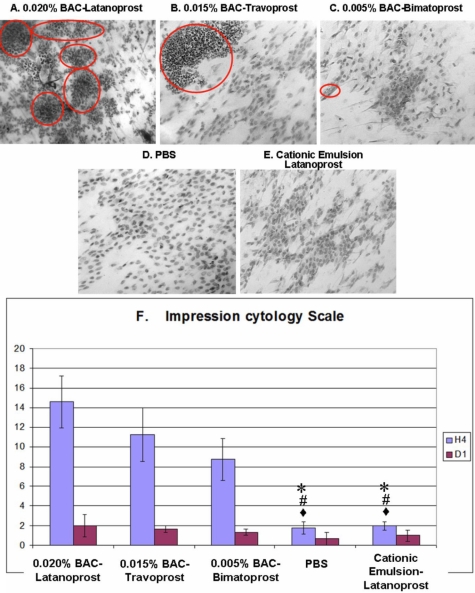
Conjunctival impression cytology stained by cresyl violet and impression cytology scale. ^BAC+^Latanoprost (**A**) induced numerous polymorphonuclear inflammatory cells as islets (red circles). ^BAC+^Travoprost (**B**) showed epithelial damage with inflammatory islets. ^BAC+^Bimatoprost (**C**) induced slight epithelial cell disorganization. PBS (**D**) and LCEm (**E**) rabbit eyes presented almost normal epithelial patterns without any obvious inflammatory cell infiltration (original size 40X). IC scale (**F**) showed that the highest toxicity was found in ^BAC+^latanoprost-, ^BAC+^travoprost-, and ^BAC+^bimatoprost-treated eyes. LCEm- and PBS-instilled groups were similar. The asterisk indicates a p<0.0005 compared with ^BAC+^latanoprost. The sharp indicates a p<0.01 compared with ^BAC+^travoprost and the filled diamond indicates a p<0.05 compared with ^BAC+^bimatoprost.

Significant infiltration of inflammatory cells was observed after the instillation of ^BAC+^latanoprost (Figure 4A, red circles) with rare and altered conjunctival epithelial cells that presented significant anisocytosis and anisonucleocytosis. No goblet cells were seen. Following instillations of ^BAC+^travoprost (Figure 4B), numerous inflammatory cell islets, principally comprising PMNs with their characteristic multilobulated nucleus, were observed. Rare goblet cells could also be observed. ^BAC+^Bimatoprost (Figure 4C) instillations induced slight anisocytosis in the epithelium with cells having normal nuclei. Some infiltration of inflammatory cells could also be observed, but without the formation of obvious inflammatory cell islets. The number and morphology of goblet cells remained normal. IC specimens from rabbit eyes instilled with PBS or LCEm (Figure 4D,E) showed an almost homogeneous and regular cell layer, with flat and regular epithelial cells and a normal nuclear:cytoplasmic ratio. A few lymphocytes were scattered among the epithelial cells; however, there were no obvious patches of PMN infiltration.

Analysis of the IC morphology score at H4 (Figure 4F) revealed that the highest IC scores were found in ^BAC+^latanoprost- and ^BAC+^travoprost-instilled groups (p<0.0005 for ^BAC+^latanoprost; and p<0.01 for ^BAC+^travoprost when compared with either PBS- and LCEm-instilled groups). The ^BAC+^bimatoprost group had a moderately high IC score at H4, which was significantly higher than those of the PBS- and LCEm-instilled groups (p<0.05 for the two groups). For the PBS- and LCEm-instilled groups, low scores were reported with no statistical difference at H4. At D1, all groups had IC scores that returned to low levels without any statistically significant differences between them.

**Figure 5:**
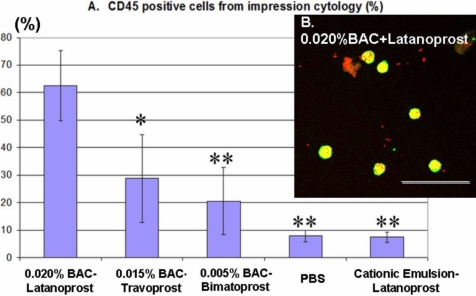
Conjunctival imprints evaluated by flow cytometry for CD45-positive cells after multiple instillations at H4, and assessed after cytospin centrifugation. The inflammatory marker clearly showed the highest expression in BAC-containing eye drops (**A**). PBS- and LCEm- instilled groups had only a basal level of expression. CD45-positive cells (green) after instillations of ^BAC+^latanoprost (**B**) were viewed after propidium iodide staining (red) and cytospin centrifugation. The scale bar indicates 50 μm. The asterisk indicates a p<0.05 compared with ^BAC+^latanoprost-instilled group. The double asterisk indicates a p<0.02 compared with ^BAC+^latanoprost-instilled group.

The impression cytology was also analyzed using FCM for CD45 (panleukocyte marker)-positive cell expression. At H4, the ^BAC+^latanoprost-treated group induced 62.63±12.76% of CD45-positive cells (p<0.05 when compared with the ^BAC+^travoprost-treated group and p<0.02 when compared with the other three groups). The ^BAC+^travoprost- and ^BAC+^bimatoprost-treated groups had 28.74±15.95% and 20.54±12.28% CD45-positive cells, respectively (Figure 5A). In PBS-instilled rabbits, approximately 8.00±2.08% of the cell population was CD45-positive, and LCEm induced only 7.39±1.83% of CD45-positive cells. There was no significant difference between these two groups. These CD45-positive cells were mostly inflammatory cells, as demonstrated by the cytospin centrifugation for the ^BAC+^latanoprost (Figure 5B) group.

## Discussion

Recent in vivo studies performed in NZW rabbits in our laboratory have demonstrated the role of cationic emulsions in reducing ocular cytotoxicity induced by quaternary ammonium compound (QAC)-containing solutions [19]. After establishing the optimal methodology and observation time required for obtaining the best and maximal information from this acute repeated-instillations model, experiments examining the effects of the new LCEm formulation were performed. Repeated instillations of the LCEm formulation onto the ocular surface of the rabbit did not induce any obvious changes of the ocular surface microstructures and the expression pattern of inflammatory markers, which presented similar results to the PBS-treated group. Our data are in accordance with previous studies concluding that BAC-containing latanoprost, travoprost, and bimatoprost eye drops were cytotoxic for the cornea/conjunctiva tissues, at levels related to the concentration of BAC [6-9]. Conversely, other in vitro or in vivo studies showed that two preservative-free PG solutions (^BAC-^travoprost Z or ^BAC-^tafluprost) did not induce any ocular surface side effects, i.e., conjunctival cytotoxicity, apoptosis, or necrosis [6,12]. BAC-free Travoprost Z showed significantly less toxicity than ^BAC+^latanoprost [26] in vitro, with no detectable loss of epithelial tight junctions [27]. Bathing rabbit eyes for 3 min with ^BAC-^travoprost Z did not cause corneal epithelial toxicity reactions. However, eyes treated with ^BAC+^latanoprost induced superficial cell loss, as a result of the presence of high concentrations (0.02%) of BAC [28]. Once-daily dosing of ^BAC-^travoprost Z produced significantly fewer corneal changes and less conjunctival inflammation than ^BAC+^latanoprost treatment did. In fact, the changes seen with ^BAC-^travoprost Z were similar to those observed in eyes treated with artificial tears [29].

In this study, we were especially interested in cationic emulsion. This new formulation offers a new strategy for ocular drug delivery of active compounds designed for the treatment of different chronic ocular diseases, such as glaucoma, dry eye, or allergy. As these diseases require long-term treatment, eye drops preserving the sensitive ocular surface are actively sought. Herein, we demonstrated the safety of a newly developed cationic emulsion formulation containing latanoprost. Latanoprost in cationic emulsion showed no obvious ocular toxicity after multiple instillations, and was as well tolerated by the ocular surface as the negative control. The positive charge of the cationic emulsion was brought about by the very low concentration of CKC (0.005%) trapped in the oil phase (oil droplets) of the nonpreserved LCEm emulsion [16,17]. It has already been demonstrated that cationic emulsions with 0.002% CKC were not toxic for the ocular surface [19]. Moreover, compared with traditional solutions, the emulsion could optimize the ocular surface homeostasis by its oily properties, since the emulsion alone was developed as a tear substitute for dry eye symptoms (Cationorm®, Novagali Pharma SA). Similar lipidic compounds have been proposed as tear substitutes with good relief of patient symptoms and signs. Thus, this new LCEm can be used for glaucoma patients, especially for those suffering from ocular surface diseases such as dry eye or allergy.

The constant and direct deleterious influence of preservatives in the cornea and conjunctiva was largely studied in previous studies [5,6,9]. There are still debates about the efficacy of drugs, with or without preservatives. In animal models or in humans, the preservative-free prostaglandin has shown a similar penetration and pharmacokinetic properties than preserved prostaglandins [30-32]. In the present study, we also examined whether the instillation of certain eye drops influenced or altered the eye-associated immunologic system. Immunotoxicity is the discipline of toxicology studying the interactions between xenobiotics and the immune system resulting in adverse effects [33]. Long-term exposure to toxic substances in a repeated manner can interact with the ocular immune system. While immune privilege, including anterior chamber-associated immune deviation, was found to be maintained after topical ^BAC+^latanoprost applications, through observations of corneal neovascularization and corneal allograft survival in mice [34], we demonstrated here that BAC-containing eye drops can nevertheless stimulate the activation of the CALT follicle, an immunologic defense mechanism of the ocular surface, by inducing inflammatory cell infiltration and lymphatic vessel circulations. CALT is organized both diffusely and in aggregates or follicles (principally of lymphocytes) in the lamina propria [35], and was mainly observed in the tarsal and orbital conjunctiva of the upper and lower lids. In contrast to BAC-containing eye drops, the instillations of PGs without BAC, such as the new LCEm, were safe for the rabbit ocular surface, showing no activation of the CALT follicle structure, i.e., no obvious inflammatory cell infiltration was detected, just as in the PBS-instilled rabbits. Published immunohistochemistry studies have confirmed that these follicles were rich in CD20-positive B cells, accompanied by diffused CD3-positive T cells at their periphery. Apart from T/B lymphocytes, macrophages, plasma, and dendritic cells (DCs) can be found [36]. The CALT immunocytes participate in diverse functions, such as antimicrobial defense, hypersensitivity (allergy), allograft rejection, and immune tolerance mechanisms [35,36]. CALT follicles were not well explored in previous toxicological studies due to their location and the lack of pertinent tools. IVCM-CALT analysis could explore in vivo the inflammatory cell trafficking, directly recording lymphocyte movements after a drug-induced stimulation. We used the CALT follicle structure to distinguish the subclinical toxicity of eye drops more sensitively and accurately, and it could be an important criterion to be considered for the IVCM toxicity scores. Cell trafficking and signaling, chemo-attractive activities within CALT follicles during immunotoxic phenomena, remain to be elucidated.

Here, we emphasize the importance of analyzing the entire ocular surface structure when assessing a drug-induced toxicity in vivo: the cornea, limbus, conjunctiva epithelium/stroma, as well as the CALT follicles. In this study, we especially improved our previously published IVCM score [19] by including the analysis of CALT structure, thus strengthening the sensitivity of the conjunctival inflammatory/toxic reaction evaluation. The three scoring systems quantify the drug-induced ocular surface alterations: (1) the modified Draize test for clinical observation, (2) the IVCM scale for microstructural images of the cornea/conjunctiva (CALT)/limbus, and (3) the IC scores for impression cytology morphology. With the development of these ex vivo and in vivo quantified systems, we did not need to sacrifice the animals at each time point to test the ocular toxicity of a new drug, the same animal was examined over the entire observation period, thus limiting individual variations.

In conclusion, we improved and standardized a set of in vivo and ex vivo tools to explore the ocular toxicity of new drug formulations and confirmed the nontoxicity profile of a new cationic emulsion containing PG analogues. This new latanoprost cationic emulsion could be proposed for the treatment of glaucoma in the near future. The emulsion may also improve the long-term compliance of patients with less discomfort and reduced ocular toxicity, especially in patients presenting dry eye symptoms.
